# P-2245. Single cell RNA-sequencing of immune cells demonstrates differential expansion of a monocyte transcriptional state among the Sepsis Response Signature (SRS) endotypes

**DOI:** 10.1093/ofid/ofae631.2398

**Published:** 2025-01-29

**Authors:** Pierre Ankomah, Alyssa DuBois, Michael R Filbin, Nir Hacohen, Roby P Bhattacharyya

**Affiliations:** Massachusetts General Hospital, Boston, Massachusetts; Broad Institute, Boston, Massachusetts; Massachusetts General Hospital, Boston, Massachusetts; Massachusetts General Hospital, Boston, Massachusetts; Massachusetts General Hospital, Boston, Massachusetts

## Abstract

**Background:**

Immune response variation among sepsis patients leads to substantial clinical heterogeneity that makes treatment challenging. Bulk RNA-sequencing of circulating immune cells has been used in an attempt to resolve sepsis heterogeneity, and has facilitated classification of patients into different subtypes, or “endotypes”. The Sepsis Response Signature (SRS) classification comprises 3 endotypes: SRS1 represents an immunosuppressed transcriptional profile with high mortality; SRS2 a relatively immunocompetent response with low mortality, and SRS3 is a profile close to a normal immune response. However, because bulk RNA-seq does not distinguish the transcriptional contributions of different cell types, mechanistic insights from these classifications are lacking, thus limiting clinical utility. Single-cell RNA-sequencing (scRNA-seq) enables cell-specific transcriptomics and has the potential to demonstrate the cellular basis of sepsis endotypes. In this study, we performed scRNA-seq on blood from sepsis patients to investigate the differences in immune cell transcriptional states among SRS endotypes.

**Figure 1.**
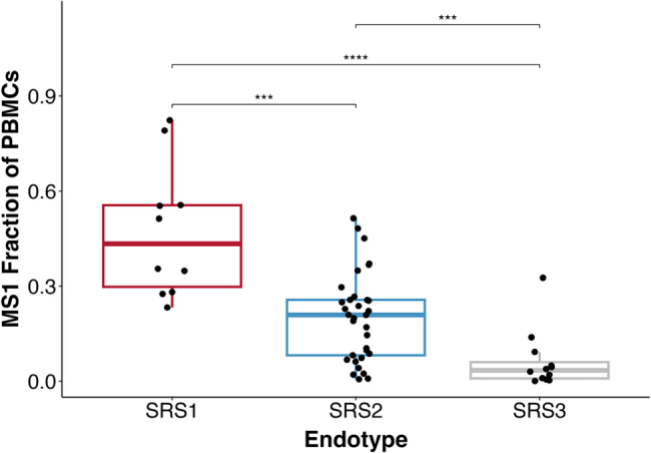
Fractional abundance of monocyte substate 1 (MS1) is highest in the Sepsis Response Signature 1 (SRS1) endotype

Fraction of MS1 cells relative to all peripheral blood mononuclear cells (PBMCs) in SRS1 (n= 10), SRS2 (n= 33), and SRS3 (n= 11) patients. Boxes show the median and inter-quartile range (IQR) for each endotype, with whiskers extending to 1.5x the IQR in either direction from the top or bottom quartile. Two-sided Wilcoxon rank-sum test with Benjamini-Hochberg correction used for comparisons; ***p<0.001, ****p<0.0001.

**Methods:**

Mononuclear immune cells were isolated from the blood of sepsis patients (n=54) at hospital presentation and the transcriptomes of ∼1500 single cells per sample were analyzed. By aggregating gene counts for each patient, we generated pseudobulked transcriptional profiles to approximate whole blood gene expression. We then leveraged the SepstratifieR machine learning framework to stratify patients into SRS endotypes and compared the proportions of immune cell transcriptional states across the endotypes.

**Results:**

A monocyte transcriptional state – monocyte substate 1 (MS1), was differentially expanded across SRS endotypes (Fig.1). MS1 abundance was significantly higher for patients in the SRS1 endotype compared to SRS2 (p< 0.001) or SRS3 (p< 0.0001).

**Conclusion:**

Identifying cellular signals underlying endotypes may improve understanding of sepsis immunobiology and open paths to precision therapy. This study demonstrates the expansion of a monocyte transcriptional state in the SRS1 endotype. MS1 cells inhibit T cell activation and proliferation, suggesting a potential mechanistic explanation for the immunosuppressive state of SRS1 patients.

**Disclosures:**

Michael R. Filbin, MD, Day Zero Diagnostics: Grant/Research Support|Quidel: Grant/Research Support

